# FACTORS ASSOCIATED WITH SUBJECTIVE COGNITIVE FUNCTION IN OLDER ADULTS WITH TYPE 2 DIABETES

**DOI:** 10.1093/geroni/igac059.2087

**Published:** 2022-12-20

**Authors:** Min Jung Kim, Cynthia Fritschi, Eunhee Cho

**Affiliations:** Yonsei University, Seoul, Seoul-t'ukpyolsi, Republic of Korea; University of Illinois Chicago, Chicago, Illinois, United States; Yonsei University, Seoul, Seoul-t'ukpyolsi, Republic of Korea

## Abstract

Subjective cognitive decline (SCD) could be an indicator of future cognitive impairment in older adults. Diabetes is a well-known risk factor of cognitive impairment, but little is known whether sleep and psychological problems common in older adults with diabetes can contribute to SCD. The study aimed to investigate whether self-reported sleep (sleep impairment and disturbance) and psychological problems (depressive symptoms and diabetes distress) were associated with subjective cognitive function in adults aged ≥ 60 years with type 2 diabetes. Sleep, depressive symptoms, and subjective cognitive function were self-reported using The Patient-Reported Outcomes Measurement Information System. Diabetes distress was assessed using the Diabetes Distress Scale. Covariates (age, sex, race/ethnicity, body mass index, and diabetes duration) were self-reported, and glycemic control (A1c) was measured using a fingerstick test kit. A total of 82 older adults were included (mean age = 68.32 ± 5.29 years, White 76.83%, female 56.1%). Multivariate regression analyses revealed that, after controlling for covariates and A1c, increased sleep impairment was associated with increased concerns on cognitive function reported by older adults (r = -.47, β = -.56). Increased depressive symptoms were also associated with decreased cognitive function perceived by older adults (r = -.44, β = -.47). Sleep disturbance and diabetes distress were not associated with subjective cognitive function. Sleep impairment and depressive symptoms were the two strongest predictors affecting subjective cognitive function. Improving sleep quality and addressing depressive symptoms could be an effective strategy to prevent SCD and potentially delay severe cognitive impairment in older adults with diabetes.

